# Advancing endoscopic traction techniques in endoscopic submucosal dissection

**DOI:** 10.3389/fonc.2022.1059636

**Published:** 2022-11-01

**Authors:** Suliman Khan, Faisal S. Ali, Saif Ullah, Xue- Huang, Hongyu Li

**Affiliations:** ^1^ The Second Affiliated Hospital of Zhengzhou University, Zhengzhou, China; ^2^ Gastroenterology, Hepatology, and Nutrition Department, University of Texas Health Science Center at Houston, Houston, TX, United States; ^3^ Department of Gastroenterology, The First Affiliated Hospital of Zhengzhou University, Changxing, Zhengzhou, China; ^4^ Department of Gastroenterology, The People’s Hospital of Changxing Country, Zhejiang Province, China

**Keywords:** gastrointestinal cancer, endoscopic submucosal dissection, traction, endoscopic training, interventional endoscopy

## Abstract

Traction techniques have emerged as a desirable “second-hand” while performing endoscopic submucosal dissection (ESD), enabling adequate visualization of submucosal tissue and vasculature, which allows for safe and efficient dissection. Multiple traction techniques have been developed over the years, and these can be broadly divided into internal and external traction techniques. This arsenal of techniques allows for traction that is personalized to the location of the lesion undergoing ESD. Mastering traction techniques requires structured training, and understanding of the benefits and pitfalls of each technique. Future research and development efforts need to focus on pathways and curriculums for trainees to master the currently available endoscopic traction techniques and provide avenues for the development of newer traction modalities.

## Introduction

Endoscopic submucosal dissection (ESD) is increasingly being adopted as the management modality of choice for early superficial gastrointestinal (GI) tumors (1–3). ESD enables resection of challenging lesions, including en-bloc resection of lesions larger than 20 mm, which translates into a higher curative resection and lower local recurrence rate (4). Despite these benefits, the applicability of ESD is limited owing to its technical complexity and potential for serious adverse events. For trainees, performing ESD entails a steeper learning curve. In the West, the applicability of ESD is further limited by lack of infrastructure to deal with the numerous obstacles faced by endoscopists when attempting to establish an ESD center (5, 6). Nonetheless, the techniques and tools for ESD continue to advance and evolve, predominantly in Japan and China.

Over the years, ESD has seen advancements in the approach to visualization and identification of the submucosal layer, such as use of a transparent cap and reduplicative submucosal injection before proceeding further with resection (7). However, the supporting capacity of transparent cap is limited, and repeated injections prolong procedure time. In recent years, tissue traction has emerged as an appealing “second-hand” while performing ESD (8). Inspired by the surgical pull and push techniques, traction not only provides clear view of submucosal tissue and vessels, but also provides adequate tissue tension during ESD. Traction techniques have been evolving since they first came to limelight. The various traction techniques currently in practice have advantages and pitfalls, depending on the location and size of the lesion (9).

**Figure 1:**
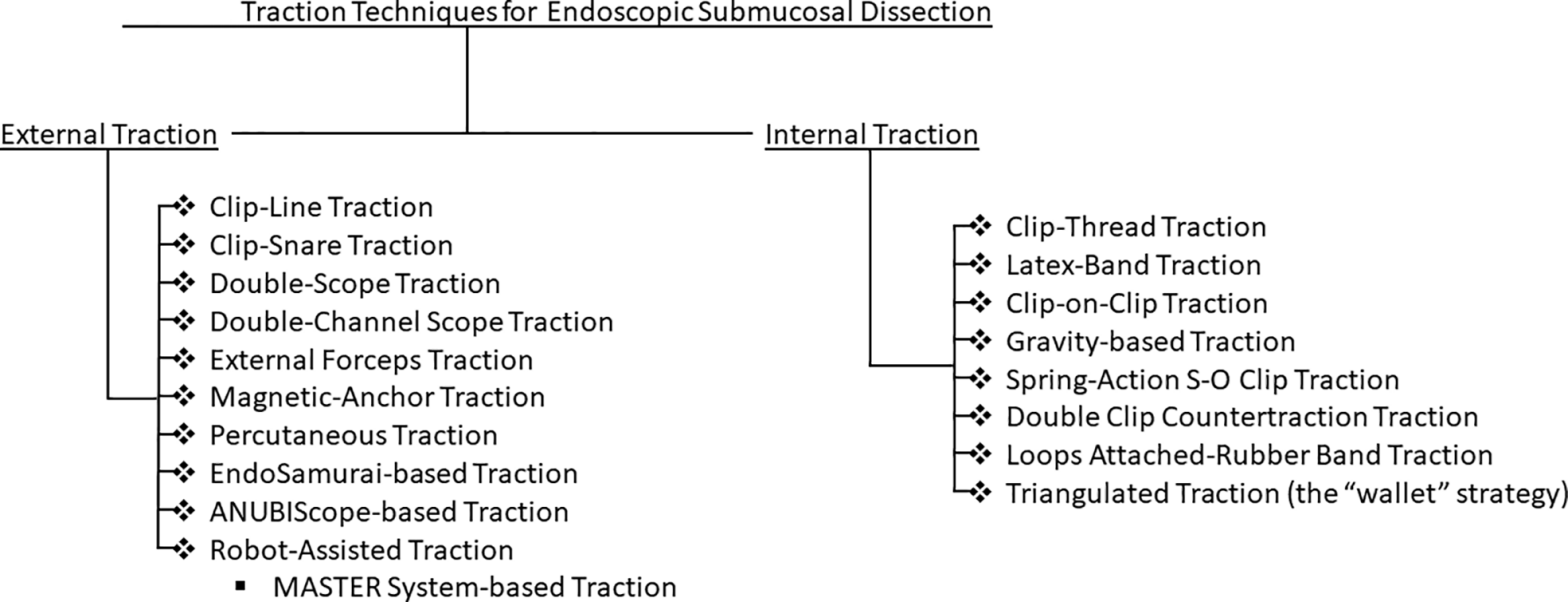
Flowchart of explored endoscopic traction techniques for endoscopic submucosal dissection.

Traction techniques can broadly be categorized into internal and external endoscopic traction technique **(** **Figure 1** **).** This article aims to summarize the evolution and characteristics of traction techniques, while highlighting the pros and cons of various approaches to traction.

## External traction techniques

Early in the course of endoscopic mucosal resection (EMR), double-scope traction, percutaneous traction, and magnetic anchor traction techniques were employed for the resection of early gastric cancer in human and animal experiments (10). Subsequently, these traction modalities evolved and were implemented to improve the feasibility of ESD.

### Percutaneous technique

The percutaneous-traction technique has mostly been utilized in animal trials (11). A laparoscopic port with a trocar is inserted directly into the gastric lumen after endoscopic determination of the proper port position. Snare forceps are subsequently inserted through the gastric port to grasp and pull the lesion to advance ESD, providing traction independent of the endoscope. The percutaneous-traction method carries a high risk of pneumoperitoneum and peritonitis. The incidence and severity of delayed adverse events also remains to be studied. Furthermore, the percutaneous-traction method is not advisable for lesions of the esophagus, the anterior gastric wall, lesions located in the high fundus of the stomach, and colorectal lesions, as this may increase the risk of adverse events.

### Magnetic-anchor technique

**Figure 2:**
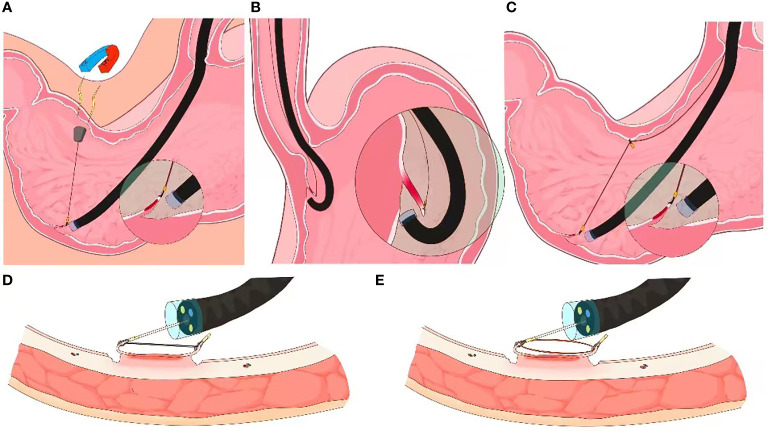
External endoscopic resection techniques. **(A)** Schematic of magnet-anchor traction technique ((from Gotoda et al). **(B)** Schematic of clip-line traction technique. **(C)** Schematic of pulley clip-line traction technique **(D, E)** Schematics of clip-snare traction technique.

Two prospective clinical trials have demonstrated the feasibility of magnetic-anchor-guided ESD of gastric lesions (12, 13). After placing an internal magnetic anchor at the target lesion, traction can be applied by a high-power electromagnet placed outside the body **(** **Figure 2A** **).** As one would expect, the magnetic force and consequently, the traction generated by this technique, depends on the patient’s abdominal wall thickness. The adverse effect profile of deploying a magnetic traction system for ESD also needs to be further elucidated to better understand its clinical utility.

### Clip-line technique

The most widely used and simplest traction techniques employ endoscopic clips. Since 2008, a variety of clip traction techniques have emerged (14, 15).

Jeon et al’ first described the clip-line traction technique in 2009 (16). A clip with dental floss or suture attached to its tail end is mounted on the endoscope. The clip is then advanced with the endoscope and deployed at the desired edge of the luminal lesion; controlled traction is achieved as the endoscopy technician or assistant manually pulls on the free end of the thread **(** **Figure 2B** **).** This is among the preferred techniques for trainees due to its relative ease of adoptability (14). The clip-line traction technique has also been used for other endoscopic interventions, such as endoscopic submucosal excavation (ESE), endoscopic full thickness resection (EFTR), and submucosal tunneling endoscopic resection (STER), where it has been shown to reduce the procedure time. However, this technique is limited by the degree of force that can be applied to the clip-line; there is a risk of clip dislodgement with excessive traction.

The clip-line technique can be modified into a pulley traction technique by deploying a second clip at the contralateral mucosa, thereby altering the direction of traction **(** **Figure 2C** **).** The pully technique is unsuitable for lesions within the esophagus, the cardia and the pylorus of stomach (17).

### Clip-snare technique

The clip-snare traction technique entails pre-looping of a snare at the tip of the endoscope before introducing it into the GI tract. First demonstrated by Yoshida et al. and later be Ota et al., this technique is more commonly known as the clip-snare method with a pre-looping technique (CSM-PLT; **Figures 2D, E**) (18, 19). A clip is subsequently inserted through the working channel of the endoscope and attached to the mucosal flap of the lesion. The snare is then loosened, advanced forward and tightened to grasp the clip. Compared to a silk thread or a dental floss, the snare has a stable sheath to provide traction by pulling or pushing the clip in forward as well as retroflexed endoscope views. Moreover, the snare and the endoscope can be maneuvered independently for flexible movement. By deploying additional, adjacent clips, this technique can be modified to provide multi-point traction (20).

### External forceps traction technique

Imaeda et al. first demonstrated the external forceps traction technique (21). After a circular incision of the lesion, the endoscope was retrieved to introduce grasping forceps with the assistance of a second forceps passing through the accessory channel of the endoscope. The grasping forceps were then anchored at the margin of the lesion like a lock. Traction is then applied, not only by pulling or pushing but also by rotation. Furthermore, when traction is not appropriate the lesion could also be released and regressed. This method has only been attempted in cases of early rectal cancer^46^. It should be noted that it is difficult to insert and control forceps-produced traction when operating on deep-seated colonic lesions.

## Internal traction techniques

### Clip-based traction techniques

Chen et al. were among the first to demonstrate an internal traction technique; two clips were deployed on opposite ends of the lesion while performing ESD (22). Hot biopsy forceps were subsequently used to pull the small loop between two clips to achieve traction. The internal traction technique evolved thereafter in diverse forms, most notably the Sakamoto-Osada clip (S-O clip) based traction technique. The S-O clip was originally developed for colonic ESD by Sakamoto et al. (23). Subsequently, multiple variations of this system have been developed and reported, including the latex-band traction technique (a nylon thread looped with a latex band which is grasped by an endoclip; the nylon thread is maneuvered to apply traction); the clip and ring shaped thread traction technique (hemoclips with attached ring shaped thread are deployed at the oral and anal edges of the lesion; a third clip grasps the thread and is fixed to the opposite mucosal wall, creating a triangulated traction system. Alternatively, a single clip may be deployed on the desired edge of resected tissue and a second clip deployed on the contralateral mucosa to create internal traction); the loops-attached rubber band traction technique (a circular rubber band connected to many smaller nylon loops which are grasped with a clip when being deployed at the site of interest); the spring-action S-O clip technique (a 7 mm x 1.8 mm spring attached to a metal clip on one end for anchoring at the ESD site and a double nylon loop on one end to be grasped by a clip for contralateral anchoring); the double-clip counter-traction technique using a rubber band (two clips connected by a rubber band), and the wallet strategy (hemoclips with attached rubber bands are deployed at the oral and anal edges of the lesion; a third clip grasps rubber bands of both clips and is fixed to the opposite mucosal wall, creating a triangulated traction system.

**Figure 3:**
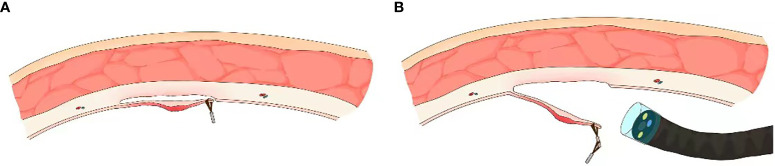
Internal endoscopic traction technique. **(A)** Schematic of S-O clip based internal traction technique (from Matsumoto et al). **(B)** Schematic of clip-on-clip method (from Nomura et al).

Nomura et al. have reported a simplified clip-on-clip method (first clip is deployed at the ESD site of interest; second clip is deployed at the base of the first clip; a third clip is deployed on the contralateral mucosa while grasping the second clip from the free space available between its grasping teeth **(** **Figure 3** **));** this technique is beneficial when performing ESD in narrow luminal regions such as the rectosigmoid colon (24). This technique also carries the advantage of providing traction independent of the endoscope. However, it is difficult to control the traction direction and the stability of this technique can be questionable at times; if the clips are placed suboptimally, adequate traction cannot be applied, and an additional clip is sometimes needed.

### Gravity based traction

In recent years, data on feasibility of gravity-based traction has emerged, mostly in the context of colorectal ESD. A magnetic bead or a sinker is attached to the edge of the lesion which can be pulled by gravity. Altering patients’ position to identify the ideal site for traction has been reported as an adjunct to facilitate adequate gravity-based traction (25).

## Traction by location

### Traction in the esophagus

The esophagus is a straight narrow tube extending from the pharynx to the cardia of the stomach. It has a special anatomy without a serosal layer and moves with respiration and heartbeats. Traction devices are seldom applied in the esophagus due its narrow lumen. Whenever needed, the clip-with-line traction technique is advisable. In a multicenter randomized controlled trial, Yoshida et al’ demonstrated the superiority of the clip-line assisted ESD over conventional ESD in terms of procedure time (44.5min vs 60.5min) and should be considered the first choice for esophageal cancers≥20mm (18). The external forceps method is another option for esophageal ESD. Motohashi et al. and Hirota et al. have reported traction techniques modified from the above technique, which needs an overtube with a side-channel for forceps introduction, or an Impact Shooter^®^ mounted on the scope (25, 26). Although this requires special devices, the direction of traction can be changed by rotating the angle of the overtube. The feasibility and potential efficacy of these traction modalities needs to be validated in larger randomized trials.

### Traction in the stomach

The stomach is a J-shaped organ with varying sizes, and consists of four layers with a fibrous serosa. It is divided into 5 regions: cardia, fundus, body, antrum, and pylorus. Sufficient space and elasticity allow for a lot of traction.

Clip-line assisted ESD has been reported to significantly shorten procedure time without compromising safety while performing resection of gastric lesions (27). Further prospective, randomized and controlled study of this technique revealed that the procedure time reduction is most pronounced when performing ESD in the greater curvature of the upper or middle stomach with a low risk of perforation (28). The CSM-PLT technique has also been used for gastric ESD and has been reported to reduce procedure time compared to conventional ESD. Preliminary study of employing the clip-snare traction technique for gastroesophageal ESD has also been reported. Similarly, S-O clip-based internal traction technique has also been used and has been reported to increase procedure speed by 25% without additional risk of adverse events (29).

External forceps-based traction technique for gastric ESD proves to be cumbersome when dealing with lesions in the cardia, lesser curvature or the posterior wall of the upper gastric body due to restricted endoscopic retroflexion (30).

There are innovative traction techniques that have been introduced in the extensive space of the stomach for ESD, such as the EndoLifter and steerable grasper with a sheath (31). The former consists of a retractable grasping forceps attached to a transparent cap by a hinge that allows simultaneous grasping, retracting, and lifting of the mucosa. The steerable grasper is fixed on the endoscope with tape, and enables bidirectional rotation upto 100 degrees. Technical feasibility and safety of these traction techniques has been demonstrated in porcine stomach models where they provide dynamic and controlled traction at various locations. Further randomized trials are warranted to accelerate translation of these techniques for human use.

### Traction in the colon and rectum

The colorectum is a long structure with several angulation segments, always accompanied by peristalsis. Colorectal ESD remains challenging due to procedural complexity, long procedure time, and high adverse events.

A novel clip-line traction technique has been designed by Yamasaki et al’., which obviates the need for withdrawal and reinsertion of the endoscope; a thread is tied to the teeth of a clip which is subsequently introduced into the lumen through the working channel of an endoscope. This technique has been utilized in a randomized controlled trail and was reported to significantly shorten procedure time compared with conventional-ESD (30). However, for lesions in the proximal colon, the efficacy of this technique dampened; it was difficult to control the force of traction applied to tissue. Yamada et al. used the CSM-PLT traction technique in a small number of patients for colorectal ESD; compared to conventional ESD, CSM-PLT reduced mean procedure time (31).

Multiple S-O clip-based traction techniques have been successfully utilized for traction during colorectal ESD, including the loops-attached rubber band traction technique, the spring-action S-O clip technique, the latex-band traction technique, the double-clip counter-traction technique, as well as the wallet strategy (both with rubber bands and ring-shaped thread). Employing the ring shaped thread for triangular traction has been shown to reduce dissection time compared to conventional ESD in a randomized trial (80 versus 130 minutes) by Mori et al. (32). As previously mentioned, the clip-on-clip traction technique is beneficial when performing ESD in narrow luminal regions such as the rectosigmoid colon (33).

## Conclusion and future perspective

ESD is a landmark advancement in therapeutic endosocpy and continues to rapidly evolve. The steep learning curve, long procedure time, and potential for serious adverse events have been challenging for trainees during the learning and application of ESD. The advent of traction techniques has decreased procedure times consistently without increasing the risk of adverse events. The various traction techniques presented above indeed facilitate the advancement and application of ESD, especially for trainees. Factors that should be considered comprehensively before choosing a traction technique include the anatomical location of lesion, the anticipated procedure time, the traction direction, the device needed (for resection as well as traction) and by extension, the type of endoscope needed, the safety of the traction technique being considered, and the cost. Ones experience with traction should also be considered. Notably, the use of traction techniques is not limited to ESD, and can be employed for emerging endoscopic technologies and other anatomical sites. Future research may focus on technological advancements and development of a structural curriculum for trainees which accommodates the increasing level of complexity of various traction techniques.

## Author contributions

Study design and concept, SK, FA, and HL. Manuscript writing, FA, SU, and SK. Data collection and analysis, XH, FA, and HL. Critical revision of the manuscript, HL. All authors contributed to the article and approved the submitted version.

## Conflict of interest

The authors declare that the research was conducted in the absence of any commercial or financial relationships that could be construed as a potential conflict of interest.

## Publisher’s note

All claims expressed in this article are solely those of the authors and do not necessarily represent those of their affiliated organizations, or those of the publisher, the editors and the reviewers. Any product that may be evaluated in this article, or claim that may be made by its manufacturer, is not guaranteed or endorsed by the publisher.
